# Robotic Transanal Minimally Invasive Surgery (R-TAMIS): A Systematic Review

**DOI:** 10.1007/s11701-026-03229-4

**Published:** 2026-02-23

**Authors:** Joachim Cheng En Ho, Clement Jing Cheng Yong, Aryan Raj Goel, Ayan Bin Rafaih, Irshad Shaikh, Muhammad Rafaih Iqbal

**Affiliations:** 1 College of Medicine and Veterinary Medicine, University of Edinburgh, Edinburgh, UK; 2 UCL medical school, University College London, London, UK; 3 St Richard’s Hospital, University Hospitals Sussex NHS Foundation Trust, Chichester, UK; 4 Aitchison College, Education Department, Lahore, Pakistan; 5 Norfolk and Norwich University Hospitals NHS Foundation Trust, Norwich, UK; 6 University of East Anglia, Norwich, UK; 7 Mid and South Essex NHS Foundation Trust, Basildon, UK

**Keywords:** Robotic surgery, Transanal minimally invasive surgery, Rectal neoplasms, Local excision, Systematic review

## Abstract

Robotic transanal minimally invasive surgery (R-TAMIS) is increasingly used for local excision of rectal lesions, offering improved precision, ergonomics and access compared with conventional TAMIS. Although early reviews demonstrated feasibility, pooled data on operative and oncological outcomes remain limited. This systematic review synthesizes contemporary evidence to provide descriptive weighted estimates of procedural efficiency, safety and short-term clinical outcomes. A systematic search of PubMed, Scopus and Web of Science through June 2025 identified studies reporting R-TAMIS outcomes in ≥ 5 patients. Extracted variables included demographics, operative metrics and oncological results. Continuous data were descriptively pooled when possible, and risk of bias was assessed using Joanna Briggs Institute and Newcastle-Ottawa tools. Owing to heterogeneity, findings were summarized qualitatively. Twenty-two studies (*n* = 437), predominantly case series, met inclusion criteria. The mean patient age was 63 years, BMI 27.5 kg/m^2^ and ASA score 2.2. Robotic systems used included da Vinci S/Si/Xi/SP and Medrobotics Flex platforms. Mean docking time was 20 min, operative duration 103 min, blood loss 18 mL and hospital stay 1.7 days. Postoperative morbidity remained low (Clavien-Dindo I–II 8%, III 1%) with reoperation and readmission rates of 2%. R0 resection was achieved in 96% of tumors, most of which are early stage (77%). R-TAMIS appears safe and feasible for local excision of early rectal tumors, with descriptive estimates suggesting low perioperative morbidity and short hospital stay. While currently more costly than laparoscopic TAMIS, efficiency gains in high-volume centers may mitigate costs. Further prospective studies are needed to better define outcomes and refine procedural indications.

## Introduction

 Rectal polyps and early rectal cancers have been treated traditionally by local excision mainly by the use of anoscope [1]. With the introduction of national bowel cancer screening programs, there has been an increasing incidence of early-stage rectal cancer detection, thus adopting organ preservation techniques [2]. Transanal endoscopic microsurgery (TEM) was developed by Dr. Gerhard Buess in 1983 [3]. This approach provided comparable results for postoperative outcomes and local recurrence as compared to total mesorectal excision [4, 5]. Atallah described transanal minimally invasive surgery (TAMIS) in 2009 [6]. TAMIS utilized the standard laparoscopic instruments through a multichannel access port. These transanal approaches offer less morbidity than the standard transabdominal approaches by avoiding stomas, reducing the length of hospital stay and improved quality of life in the form of bladder, bowel and sexual functions [1, 7].

TAMIS has become a widely accepted modality for the local excision of benign rectal polyps and early-stage rectal cancers. It offers outcomes comparable to TEM, providing minimally invasive access to narrow and confined rectal compartments while utilizing similar laparoscopic instruments, apart from the access platform [8]. With the advancements in robotic surgery, robotic TAMIS (R-TAMIS) was introduced in 2012 and has started to gain widespread adoption [9]. R-TAMIS incorporates articulated instrumentation, three-dimensional magnified visualization, motion scaling and improved ergonomics, potentially enabling more precise excisions in anatomically challenging scenarios [10].

The da Vinci surgical systems (Intuitive Surgical, Sunnyvale, California, USA) have been central to the development of R-TAMIS. Early generations, such as the Si platform, were limited by bulky arms, restricted reach and complex docking, particularly when addressing lesions farther from the anal verge or navigating curved rectal segments [11]. The subsequent Xi platform addressed many of these limitations, featuring slimmer arms, improved access and simplified docking [12]. In a cohort of 58 R-TAMIS cases, Tomassi et al. [13] reported a mean operative time of 66 min (range 17–180 min), noting that procedures performed with the Xi platform were significantly more efficient than those using the Si (38.7 vs. 78.5 min; *p* = 0.00003).

More recently, the Single-Port (SP) da Vinci platform has been introduced to further overcome the limited operative field associated with bulky arms. Early reports suggest that the SP system may offer advantages such as shorter docking times due to single-docking procedures, while maintaining outcomes comparable to TEM and other TAMIs platforms [9].

Despite the growing popularity of R-TAMIS, high-quality randomized controlled trials comparing R-TAMIS with TEM or conventional TAMIS are still lacking. Contemporary real-world practice in transanal management of rectal neoplasia remains heterogeneous in terms of indications, platform choice, and follow-up pathways, reinforcing the need for focused evidence synthesis on newer robotic approaches [14]. While two previous systematic reviews published have summarized early experiences, several more recent studies provide new insights into procedural efficiency, lesion accessibility and clinical outcomes. An updated synthesis is therefore warranted to capture the latest evidence and inform surgeons and centers considering the adoption of R-TAMIS.

## Methods

### Protocol registration

This systematic review was registered with PROSPERO (ID: CRD42025627874) and conducted in accordance with the PRISMA guidelines [17].

### Eligibility criteria

The eligibility criteria were set out as follows:

1. Primary studies up to June 2025 utilizing robotic platforms for TAMIS procedures, including interventional and observational studies and case series.
2. Case series retrieved had to include more than five patients.
3. All primary studies retrieved had to present R-TAMIS data such as patient characteristics and operative outcomes, even if combined with other platforms or procedures.

The exclusion criteria were:

1. Articles in the format of technical notes, reviews, abstract-only articles, conference abstracts, case reports, case series with fewer than five patients, video articles without extractable data.
2. Articles reporting other procedures such as total mesorectal excisions (TME) or low anterior resections (LAR).
3. Articles containing unextractable R-TAMIS data.
4. Articles not in the English language.

### Sources and search strategy

Three databases—Scopus, Web of Science and PubMed, were searched up to June 2025. Keywords included “robotic”, “robot* assisted”, “transanal”, “minimally invasive surger*” and “TAMIS”. In PubMed, MeSH Major Topic phrases were used. To exclude TME-related articles, the Boolean operator “NOT” was combined with “TME OR total mesorectal excision”. Automated filters were applied to limit results to English-language articles and exclude reviews, editorials, meeting abstracts and videos.

### Study Selection and screening

After manually removing duplicates, two authors (J.C.E.H. and C.Y.) independently screened titles and abstracts under the guidance of the senior author (M.R.I.). Full-text screening was subsequently performed by the same authors. Any disagreements were resolved through discussion with the senior author.

R-TAMIS was defined as the use of a robotic surgical platform in combination with a transanal access port for local excision of rectal lesions. R0 resection was defined as complete excision with microscopically negative margins as reported by each study’s pathology assessment. Complications were categorized as intraoperative or postoperative. Postoperative complications were graded using the Clavien–Dindo classification where reported. Studies not reporting CD grades were included, and events were recorded as reported.

### Data extraction and synthesis

Data were categorized into several domains: patient and study information, operative outcomes and oncological outcomes. Patient and study information included first author, title, year, country, study design, sample size, platform, age, body mass index (BMI), sex and American Society of Anesthesiologists (ASA) score. Operative outcomes included intraoperative and postoperative variables such as docking time, total operative time, length of hospital stay, estimated blood loss (EBL), reoperation and readmission rates. Oncological outcomes included distance from the anal verge (DAV), histopathology, pT staging, margin status, follow-up duration and recurrence rates.

Quantitative synthesis was limited by heterogeneity among robotic platforms (da Vinci S, Si, Xi, SP and Medrobotics Colorectal Drive), indication (adenocarcinoma vs. non-adenocarcinoma), distance from anal verge, and the lack of data extraction on tumor size. The quantitative analysis of oncological outcomes such as recurrence and complication was further restricted by the varying follow-up duration. Heterogeneity may have been further influenced by factors not thoroughly extracted, including surgeon experience, learning curve, patient positioning and individual study reporting standards.

Continuous variables—including patient age, BMI, ASA score, docking time, operative time, length of hospital stay, EBL, DAV and follow-up duration were summarized as descriptive weighted estimates using study sample size as weight, where possible. Other outcomes were reported qualitatively. Continuous variables were retrieved as reported, most commonly as means with standard deviations (SD). Where individual patient data were available, means and SDs were calculated directly. For studies reporting medians with ranges or interquartile ranges (IQR), means and SD were estimated using the formulas proposed by Wan et al. [18]. In studies reporting only means with ranges, medians were derived from the reported means to allow subsequent calculation of SD using the same method. Extracted and estimated values are presented separately after the originally reported data. These descriptive weighted estimates summarize central trends across studies and are not intended as formal meta-analytic effect sizes.

In two studies [19, 20], DAV was reported differently: one as distance from the anorectal ring and the other as distance from the dentate line. To standardize these measures to DAV, mean anatomical measurements described by Nivatvongs et al. [21] were applied. Specifically, 4.2 cm was added when the distance was measured from the anorectal ring and 2.1 cm when measured from the dentate line.

### Risk of bias assessment

Risk of bias was assessed independently by J.C.E.H. and C.Y., with unresolved disagreements reviewed by the senior author (M.R.I.). The JBI critical appraisal tool [22] was used for case series, while the Newcastle-Ottawa Scale [23] was used for cohort studies.

## Results

### Study selection

**Fig. 1:**
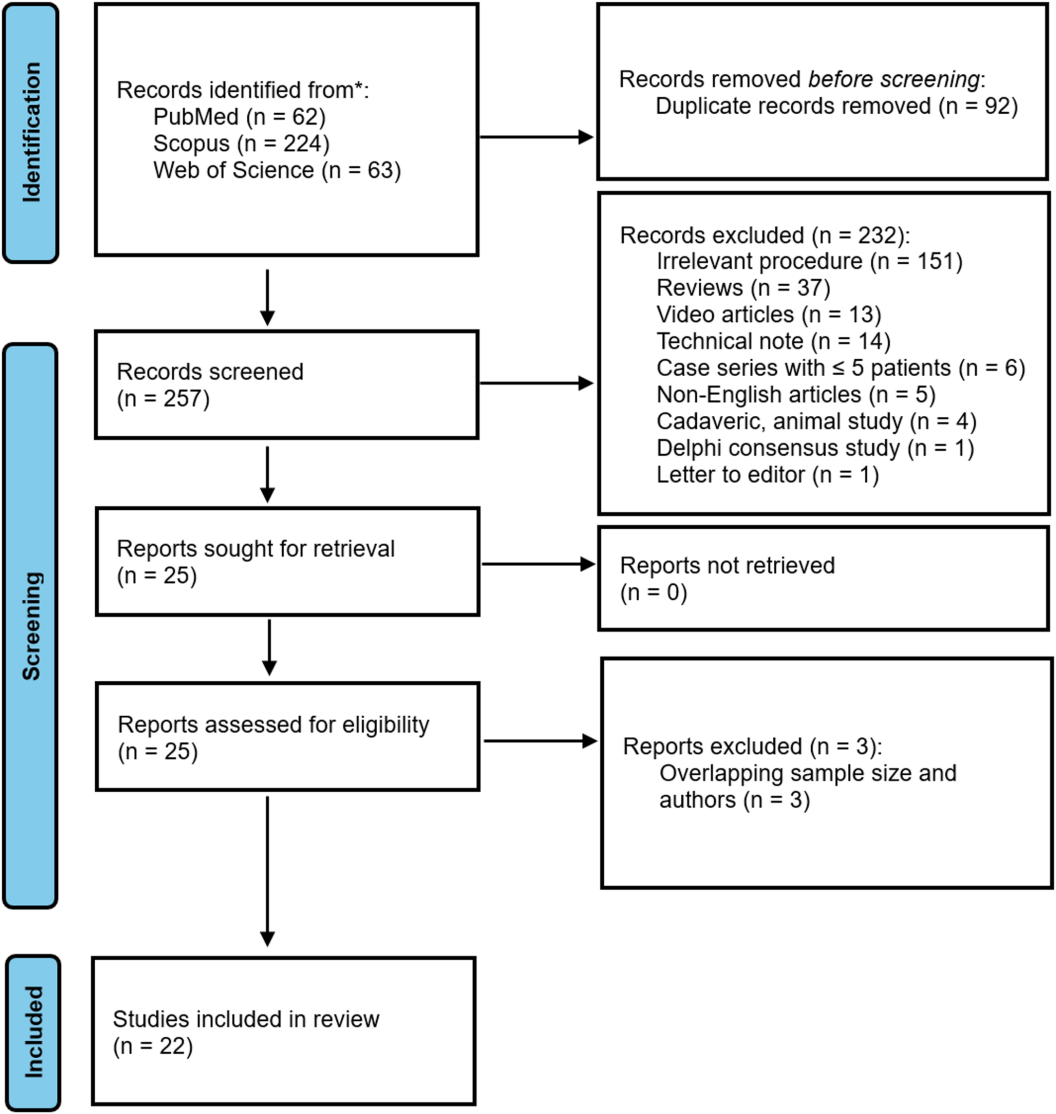
PRISMA flow diagram

A total of 349 records were identified through the initial database search (62 from PubMed, 224 from Scopus and 63 from Web of Science). After removal of duplicates (*n* = 92), 257 records underwent title and abstract screening. Of these, 232 articles were excluded for not meeting eligibility criteria (they were largely reviews, case reports, video articles or technical notes). The remaining 25 full-text articles were assessed, with 22 studies ultimately included in the qualitative synthesis. Figure 1 shows the PRISMA flow diagram.

### Study characteristics

A total of 22 studies were included in this review, encompassing 437 patients. The included studies were published between 2014 and 2024 and originated from the USA (*n* = 11), Australia (*n* = 2), Taiwan (*n* = 3), Korea (*n* = 2), UK (*n* = 2), Mexico (*n* = 1), Singapore (*n* = 1), Spain (*n* = 1) and Switzerland (*n* = 1). Most studies were case series (*n* = 19), while 3 were retrospective cohort studies.

Robotic platforms varied across studies: da Vinci S (*n* = 1), Si (*n* = 8), Xi (*n* = 12), SP (*n* = 4) and Medrobotics Flex Colorectal Drive (*n* = 2). Several studies included multiple platforms for separate patient cohorts, including Atallah et al. [24], Paull et al. [25], Tomassi et al. [13] and Yao et al. [26].

Sample sizes ranged from 5 to 58 patients per study, with a median of 17.5 patients Table 1.

**Table 1:** Study characteristics

First author	Year	Country	Study design	Sample size	Platform
Atallah [24]	2015	USA	Case series	9^a^	S/Si
Baker [27]	2020	Australia	Case series^b^	11	Xi
Ferrari [28]	2024	USA	Case series	31	SP
Fok [29]	2022	Australia	Case series	5	Flex
Guraieb-Trueba [30]	2021	Mexico	Case series	5	Si
Hompes [31]	2014	Switzerland, UK, USA	Case series	16	Si
Huang [32]	2019	Taiwan	Case series	23	Xi, *n* = 2; Si, *n* = 21
Keller [20]	2024	USA	Retrospective cohort	50	SP
Kim [33]	2024	Korea	Case series	9	SP
Lee [10]	2018	USA	Retrospective cohort	19	Xi/Si
Liu^c^ [19]	2018	USA	Case series	34	Xi
Liu^c^ [34]	2022	USA	Case series	28	Xi
Lo [35]	2021	USA	Case series	16	Xi
Ngu [12]	2018	Singapore, Taiwan	Case series	6	Xi
Paull [25]	2019	USA	Retrospective cohort	21	Xi, *n* = 10; Flex, *n* = 11
Piozzi [36]	2024	UK	Case series	20	Xi
Ruiz [37]	2017	Spain	Case series	9	Si
Schwab [38]	2022	USA	Case series	10	Xi
Tomassi [13]	2019	USA	Case series	58	Xi, *n* = 18; Si, *n* = 30
Wassef [39]	2022	USA	Case series	19	N/A
Woo [40]	2024	Korea	Case series	14	SP
Yao [26]	2020	Taiwan	Case series	24	Si, *n* = 7; Xi, *n* = 17

^a^Total sample size in this study [24] was 18, but only 9 met the inclusion criteria

^b^This case series was the only prospective study

^c^These two studies had the same author but were included as they reported separate patient cohorts from different years

S, Si, Xi and SP refer to the da Vinci systems S, Si, Xi and Single-Port respectively. Flex refers to Medrobotics Flex Colorectal Drive

### Patient characteristics

Patient demographics and baseline characteristics are summarized in Tables 2 and 3. Table 2 reports the characteristics as presented in the original studies, while Table 3 shows extracted or estimated continuous variables. Across the included studies, the descriptively weighted mean age (*n* = 360) was 63.1 ± 13.2 years, mean BMI (*n* = 363) was 27.5 ± 6.2 kg/m^2^ and mean ASA score (*n* = 274) was 2.2 ± 0.63. These data represent a cohort of predominantly middle-aged adults with mild to moderate systemic comorbidity.

**Table 2:** Patient characteristics as reported in the original studies*

First author	Sample size	Age, years	Sex, % male	BMI, kg m^–2^	ASA, *n*
Atallah [24]	9	65	44	26	N/A
Baker [27]	11	N/A	55	N/A	N/A
Ferrari [28]	31	61 ± 13.3	61	30 ± 7.4	ASA II, 11 ASA III, 18 ASA IV, ≥ 1 ASA X, N/A
Fok [29]	5	67	100	N/A	N/A
Guraieb-Trueba [30]	5	56.2, 34–79^b^	40	N/A	N/A
Hompes [31]	16	68^a^, 38–86^b^	50	26^a^, 22–29^b^	ASA I, 2 ASA II, 13 ASA III, 1
Huang [32]	23	61^a^, 33–85^b^	48	23.5^a^, 17.9–31.8^b^	ASA I, 3 ASA II, 16 ASA III, 4
Keller [20]	50	63.36 ± 12.82	52	27.84 ± 4.54	ASA II^b^, ASA I–IV^b^
Kim [33]	9	N/A	56	N/A	ASA I, 5 ASA II, 4
Lee [10]	19	67.2	67	29.52	ASA 2.75^d^
Liu [19]	34	63 ± 10	67	29.4 ± 5.9	ASA II, 13 ASA III, 20 ASA IV, 1
Liu [34]	28	62.2 ± 9.8	71	30 ± 6.3	ASA I, 1, ASA II, 16 ASA III, 10 ASA IV, 1
Lo [35]	16	63	63	N/A	N/A
Ngu [12]	6	60^a^, 51–78^b^	33	26.75	ASA II, 5 ASA III, 1
Paull [25]	10 (Si)	56 ±13.3	60	26.88 ± 5.3, 17.8–36.7^b^	ASA 1.8 ± 0.8^d^
	11 (Flex)	55 ± 16.7	64	27.7 ± 6.7, 19.75–37.93	ASA 2.1 ± 0.5^d^
Piozzi [36]	20	69.5^a^, 62–77.7	60	31^a^, 23.8–28.7^c^	ASA II, 6 ASA III, 14
Ruiz [37]	9	72.26, 58–86^b^	44	N/A	N/A
Schwab [38]	10	58^a^, 50–65^c^	50	24.7^a^, 23.8–28.7^c^	ASA I, 2 ASA II, 7 ASA III, 1
Tomassi [13]	58	N/A	N/A	27.8, 22–43.4^b^	N/A
Wassef [39]	19	69.05, 47–88^b^	42	N/A	ASA II, 13 ASA III, 6
Woo [40]	14	72^a^, 54–85^b^	79	22.6^a^, 20–29^b^	ASA II, 10 ASA III, 4
Yao [26]	24	59.4^a^, 34–83^b^	58	23.9 ± 4.1	ASA II, 23 ASA III, 1

*Values are reported as I the original studies, except sex, % male, which has been rounded to two significant figures

^a^median

^b^range

^c^Interquartile range

^d^mean ± standard deviation reported by individual studies

BMI = Body mass index; ASA = American Society of Anesthesiologists; N/A = Not available

**Table 3:** Extracted or estimated continuous patient data

First author	Sample size	Age, years	BMI, kg m^–2^	ASA
Atallah [24]	9	64.8 ± 17.2	26.2 ± 4.5	N/A
Baker [27]	11	69.8 ± 11.8	26.2 ± 5.6	N/A
Ferrari [28]	31	61.0 ± 13.3	30.0 ± 7.4	N/A
Fok [29]	5	67.0 ± 10.1	N/A	N/A
Guraieb-Trueba [30]	5	56.2 ± 17.5	25.0 ± 3.0	N/A
Hompes [31]	16	65.0 ± 13.6^a^	28.3 ± 4.8^a^	1.9 ± 0.4
Huang [32]	23	60.0 ± 13.5^a^	24.2 ± 3.6^a^	2.0 ± 0.5
Keller [20]	50	63.4 ± 12.8	27.8 ± 4.5	2.3 ± 0.7^a^
Kim [33]	9	54.3 ± 14.1	26.1 ± 3.7	1.4 ± 0.5
Lee^b^ [10]	19	67.2	29.5	2.8
Liu [19]	34	63.0 ± 10.0	29.4 ± 5.9	2.6 ± 0.5
Liu [34]	28	62.2 ± 9.8	30.0 ± 6.3	2.4 ± 0.6
Lo [35]	16	63.0 ± 15.0	N/A	N/A
Ngu [12]	6	62.3 ± 10.5^a^	N/A	2.2 ± 0.4
Paull [25]	10 (Si)	57.0 ± 13.3	26.9 ± 5.3	1.8 ± 0.9
	11 (Flex)	56.0 ± 16.7	27.7 ± 6.7	2.1 ± 0.6
Piozzi [36]	20	69.7 ± 12.5^a^	29.5 ± 12.4^a^	2.7 ± 0.5
Ruiz [37]	9	72.7 ± 8.6	N/A	N/A
Schwab [38]	10	57.7 ± 12.9^a^	25.7 ± 4.2^a^	1.9 ± 0.5
Tomassi [13]	58	N/A	27.8 ± 5.4	N/A
Wassef [39]	19	69.1 ± 11.1^a^	N/A	2.3 ± 0.5
Woo [40]	14	69.1 ± 8.7^a^	23.4 ± 2.9^a^	2.3 ± 0.5
Yao [26]	24	59.0 ± 12.6^a^	23.9 ± 4.1	2.0 ± 0.2

^a^Estimated using formulas from Wan et al. [18]

^b^Standard deviation could not be calculated due to unavailable range or interquartile range

BMI = Body mass index; ASA = American Society of Anesthesiologists; N/A = Not available

### Operative outcomes

Tables 4 and 5 summarize the operative findings across the included studies. Table 4 details intraoperative and postoperative events as originally reported, whereas Table 5 consolidates extracted or estimated continuous data. Intraoperative complications were uncommon and largely minor, such as limited pneumoperitoneum or access-related issues, which were managed conservatively. Postoperative morbidity was generally low, with most events classified as Clavien-Dindo grade I–II (*n* = 30/386, 8%) and only a few isolated grade III complications (*n* = 5/386, 1%). Reoperations and readmissions were rare (*n* = 8/351, 2% and 8/325, 2% respectively).

The pooled estimates showed a mean docking time (*n* = 44) of 19.9 ± 19.0 min, total operative time (*n* = 437) of 102.8 ± 55.2 min, mean hospital stay (*n* = 369) of 1.7 ± 2.2 days and mean estimated blood loss (*n* = 127) of 17.6 ± 22.6 mL.

**Table 4:** Operative outcomes as reported in the original studies

First author	Sample size	Complications		Docking time, minutes	Total operative time, minutes	Length of hospital stay, days	Estimated blood loss, mL	Unplanned	
		Intraoperative	Postoperative, *n*					Re-op	Readmissions
Atallah [24]	9	1 abdominal distention due to pneumodissection into abdominal cavity	CD1, 1 CD2, 1	N/A	124	0.8	35	N/A	N/A
Baker [27]	11	1 bleed requiring endoscopic clipping	N/A	N/A	N/A	N/A	N/A	0	0
Ferrari [28]	31	N/A	CD1, 1 CD2, 2	N/A	106 ± 42	N/A	N/A	0	2
Fok [29]	5	N/A	0	N/A	143	N/A	N/A	0	0
Guraieb-Trueba [30]	5	1 peritoneal access due to lesion at RSJ	0	N/A	85, 60–195^b^	N/A	N/A	0	0
Hompes [31]	16	1 Pneumoperitoneum conservatively managed	CD2, 2	36^a^, 18–75^b^	108^a^, 40–180^b^	1.3^a^, 0–4^b^	N/A	0	0
Huang [32]	23	0	0	N/A	107^a^, 15–220^b^	3.0^a^, 1–10^b^	< 30	0	0
Keller [20]	50	0	CD2, 2 CD3, 1	N/A	104 ± 65	1^b^, 0–3^b^	N/A	0	0
Kim [33]	9	0	0	N/A	68	1.7	10.2	0	0
Lee [10]	19	N/A	CD2, 1	N/A	102^a^, 22^c^	0.4^a^, 0.5^c^	5^b^, 5^c^	0	0
Liu [19]	34	0	CD2, 1	25 ± 14^d^	100 ± 70	1.2 ± 0.8	N/A	N/A	N/A
Liu [34]	28	1 intraoperative hypotension	CD2, 1	N/A	132 ± 47	N/A^e^	N/A	0	1
Lo [35]	16	0	CD2, 2 CD3, 1	N/A	87, 55–110^c^	0, 0–1^c^	17.5, 8.75–20^c^	4	N/A
Ngu [12]	6	N/A	N/A	N/A	106.5^a^, 69–217^b^	4.2 ± 1.3, 1–8^c^	N/A^e^	0	0
Paull [25]	10 (Si)	1 fractured mass; 1 proctotomy	CD3, 1	N/A	167 ± 84, 101–361^b^	N/A	37.5 ± 38.3, 5–100^b^	1	0
	11 (Flex)	1 case aborted as patient refused anterior resection and was awaiting endoscopic submucosal dissection	0	N/A	110 ± 40, 55–180^b^	N/A	9.5 ± 13.6, 5–50^b^	0	0
Piozzi [36]	20	0	CD1, 4 CD2, 1	N/A	90^b^, 60–113^c^	2^a^, 1–3^c^	0^a^, 0–12.5^c^	0	2
Ruiz [37]	9	0	CD1, 1 CD2, 2	N/A	71.9, 40–120^b^	2, 1–5^b^	39.8, 20–50^b^	0	0
Schwab [38]	10	N/A	N/A	N/A	76^b^, 51–101^c^	1^a^, 0–1^c^	5^a^, 5–50^c^	1	N/A
Tomassi [13]	58	N/A	CD1, 1 CD2, 3 CD3, 2	N/A	66, 17–180^b^	N/A	N/A	2	2
Wassef [39]	19	0	CD1, 5 CD2, 1	N/A	98, 66–134^b^	15 SD, 4 ON	N/A^e^	N/A	N/A
Woo [40]	14	0	CD1, 1	7^a^, 3–12^b^	175^a^, 70–210^b^	5^a^, 3–11^b^	20^a^, 0–50^b^	0	0
Yao [26]	24	0	N/A	N/A	130^b^, 60–240^b^	4.6^a^, 3–11^b^	N/A^e^	N/A	N/A

^a^median

^b^range

^c^Interquartile range

^d^includes undocking time

^e^Estimated blood loss in these patients were minimal

Re-op = Reoperations; CD = Clavien-Dindo; N/A = Not available; RSJ = Rectosigmoid junction; SD = Same day; ON = Overnight

**Table 5:** Extracted or estimated continuous operative outcomes

First author	Sample size	Docking time, minutes	Total operative time, minutes	Length of hospital stay, days	Estimated blood loss, mL
Atallah [24]	9	N/A	123.9 ± 50.6	0.9 ± 1.2	35.6 ± 19.9
Baker [27]	11	N/A	64.1 ± 22.6	1.5 ± 0.9	N/A
Ferrari [28]	31	N/A	106.0 ± 42.0	0.1 ± 0.5	N/A
Fok [29]	5	N/A	142.8 ± 55.1	0.6 ± 0.8	N/A
Guraieb-Trueba [30]	5	6.6 ± 2.8	97.0 ± 49.6	1.6 ± 1.2	N/A
Hompes [31]	16	41.3 ± 16.1^a^	109.0 ± 39.6^a^	1.7 ± 1.1^a^	N/A
Huang [32]	23	N/A	112.3 ± 53.1^a^	4.3 ± 2.3^a^	N/A
Keller [20]	50	N/A	104.0 ± 65.1	1.3 ± 0.7^a^	N/A
Kim [33]	9	8.7 ± 3.3	65.6 ± 21.7	1.7 ± 0.5	9.4 ± 5.0
Lee [10]	19	N/A	102.0 ± 17.6^a^	0.4 ± 0.4^a^	5.0 ± 4.0^a^
Liu [19]	34	N/A	100.0 ± 70.0	1.2 ± 0.8	N/A
Liu [34]	28	N/A	132.5 ± 46.8	N/A	N/A
Lo [35]	16	N/A	87.2 ± 46.6	1.1 ± 2.6	17.5 ± 13.1
Ngu [12]	6	N/A	124.8 ± 57.7^a^	4.2 ± 1.3	N/A
Paull [25]	10 (Si)	N/A	167.0 ± 84.2	N/A	37.5 ± 38.3
	11 (Flex)	N/A	110.1 ± 39.9	N/A	9.5 ± 13.6
Piozzi [36]	20	N/A	87.5 ± 41.9^a^	2.0 ± 1.6^a^	4.2 ± 10.0^a^
Ruiz [37]	9	N/A	76.4 ± 26.6	2.6 ± 1.0	41.7 ± 11.1
Schwab [38]	10	N/A	76.0 ± 43.0^a^	0.7 ± 0.9^a^	20.0 ± 38.7^a^
Tomassi [13]	58	N/A	66.2 ± 44.0	0.1 ± 0.3	N/A
Wassef [39]	19	N/A	96.0 ± 18.4^a^	N/A	N/A
Woo [40]	14	7.3 ± 2.6^a^	157.5 ± 38.5^a^	6.0 ± 2.3^a^	22.5 ± 14.6^a^
Yao [26]	24	N/A	139.8 ± 46.2^a^	5.8 ± 2.1^a^	N/A

^a^Estimated using formulas from Wan et al. [18]

N/A = Not available

### Oncological outcomes

Oncological findings are summarized in Tables 6 and 7. Table 6 presents the oncological outcomes as reported in the original studies, while Table 7 shows extracted or estimated continuous variables. According to the studies, the pooled mean tumor DAV (*n* = 416) was 8.2 ± 3.5 cm, and the mean follow-up duration (*n* = 263) was 16.0 ± 10.9 months. 38% of histopathology showed adenocarcinoma (*n* = 167/434). Most cases represented early-stage malignancy (pT1–pT2; 77%, *n* = 128/167), and R0 resection margins were achieved in nearly all specimens (96%, *n* = 393/408). Margin status was reported in 20 studies (*n* = 412) while recurrence was reported in 15 studies (*n* = 312).

**Table 6:** Oncological outcomes as reported in the original studies

First author	Sample size	Distance from anal verge, cm	Histopathology, *n*		pT stage, *n*	Margins, % negative	Follow-up duration, months	Recurrence, *n*
			AC	Non-AC				
Atallah [24]	9	6.6	3	6	pTis, 5 pT1, 1 pT2, 1 pT3, 1	89	11.4	N/A
Baker [27]	11	N/A	2	9	pT2, 1 pT3, 1	90	N/A	1
Ferrari [28]	31	10 ± 2.6, 6–16^a^	14	17	pT1, 6 pT2, 7 pT3, 1	97	18.3 ± 14.0	0
Fok [29]	5	8.3	0	5	0	80	N/A	N/A
Guraieb-Trueba [30]	5	8.8	1	4	pT1, 1	100	22.6, 6–39^a^	1
Hompes [31]	16	8^b^, 3–10^a^	4	10	pT1, 2 pT2, 1 pT3, 1	87	N/A	N/A
Huang [32]	23	5^b^, 2–8^a^	11	12	pTis, 1 pT1, 10	91	9.6^b^	0
Keller [20]	50	4.7 ± 4.2, 2–18^a, c^	12	38	ypT0, 3 ypT1, 2 ypT2, 3 pT1/2, 4	100	N/A	N/A
Kim [33]	9	6.4	1	8	pTis, 1	100	N/A	0
Lee [10]	19	8.2^b^, 2.1^d^	5	14	pT1, 3 pT2, 2	N/A	N/A	N/A
Liu [19]	34	8.6 ± 3.6^e^	7	27^f^	pT1, 3 pT2, 3 pT3, 1	100	6.3 ± 7.0	0
Liu [34]	28	7.8 ± 3.9, 0–16^a^	8	20	pT1, 4 pT2, 4	96	23.65, 5.9–42.4^a^	0
Lo [35]	16	9 DR; 7 MR	4	12	pT1, 3 pT3, 1	100	N/A	N/A
Ngu [12]	6	5.5^b^, 5.0–10.0^a^	6	0	pCR, 6	N/A	18.2^b^, 7–39^a^	0
Paull [25]	10 (Si)	11.1 ± 3.8, 6–20^a^	2	8	N/A	100	N/A	0
	11 (Flex)	9.6 ± 3.6, 2–17^a^	1	10	N/A	100	N/A	0
Piozzi [36]	20	7.5^b^, 5–11.7^d^	17	3	pT1, 11 pT2, 5 pT3, 1	90	11.5^b^, 3.5–17.0^d^	2
Ruiz [37]	9	6.22, 4–9^a^	1	8	pT1, 1	100	18, 15–23^a^	0
Schwab [38]	10	8^b^, 7–10^d^	3	7	pT1, 2 pT2, 1	100	3–60^b^	N/A
Tomassi [13]	58	8.8, 4–14^a^	32	26	pTis, 1 pT1, 29 pT2, 1 pT3, 1	95	16.9, 11.5–27.4^a^	3
Wassef [39]	19	7, 1–15^a^	4	17^g^	N/A	100	N/A	1
Woo [40]	14	7^b^, 2–10^a^	14	0	ypT1, 1 ypT2, 2 pTis, 6 pT1, 5	93	24^b^, 12–34^a^	0
Yao [26]	24	5.9^b^, 3.5–12^a^	15	9	ypT0, 6 pTis, 3 pT1, 3 pT2, 3	100	23.6^b^, 4–45^a^	1

^a^Range

^b^Median

^c^These values were reported as distance from anorectal ring

^d^Interquartile range

^e^These values were reported as distance from dentate line

^f^Data for one histopathology outcome were unavailable in the original report

^g^Some patients in this study [39] had multiple pathology in the specimen

AC = Adenocarcinoma; N/A = Not available; DR = Distal rectum; MR = Middle rectum

.

**Table 7:** Extracted or estimated continuous oncological outcomes

First author	Sample size	Distance from anal verge, cm	Follow-up duration, months
Atallah [24]	9	6.7 ± 2.6	11.4 ± 9.0
Baker [27]	11	7.3 ± 3.1	N/A
Ferrari [28]	31	10.0 ± 2.6	17.5 ± 14.1
Fok [29]	5	7.3 ± 3.7	N/A
Guraieb-Trueba [30]	5	8.8	22.6 ± 14.0^a^
Hompes [31]	16	7.3 ± 2.0^a^	N/A
Huang [32]	23	5.0 ± 1.6^a^	N/A
Keller [20]	50	8.9 ± 4.2^b^	N/A
Kim [33]	9	6.6 ± 1.7	15.2 ± 3.7
Lee [10]	19	8.2 ± 1.7^a^	N/A
Liu [19]	34	10.7 ± 3.6^c^	6.3 ± 7.0
Liu [34]	28	7.8 ± 3.9	23.7 ± 9.1^a^
Lo [35]	16	N/A	2.7 ± 4.3
Ngu [4]	6	6.5 ± 2.0^a^	20.6 ± 12.5
Paull [25]	10 (Si)	11.1 ± 3.8	N/A
	11 (Flex)	9.6 ± 3.6	N/A
Piozzi [36]	20	8.1 ± 5.3^a^	10.7 ± 10.8^a^
Ruiz [37]	9	6.4 ± 1.7	18.0 ± 2.7^a^
Schwab [38]	10	8.3 ± 2.6^a^	N/A
Tomassi [13]	58	8.8 ± 2.3	16.9 ± 4.0
Wassef [39]	19	7.0 ± 3.8^a^	N/A
Woo [40]	14	6.9 ± 2.6	23.5 ± 6.4
Yao [26]	24	6.8 ± 2.2^a^	24.1 ± 10.5^a^

^a^Estimated using formulas from Wan et al. [18]

^b^Distances estimated using Nivatvongs et al.[21], applying a + 4.2 cm correction to approximate the distance from the anorectal ring to the anal verge

^c^Distances estimated using Nivatvongs et al. [21], applying a + 2.1 cm correction to approximate the distance from the dentate line to the anal verge

N/A = Not available

### Risk of bias

Both reviewers reached full consensus across all appraisal domains. Overall, methodological quality was excellent, with most studies (*n* = 18) graded as low risk of bias with the rest (*n* = 4) as moderate risk.

**Fig. 2:**
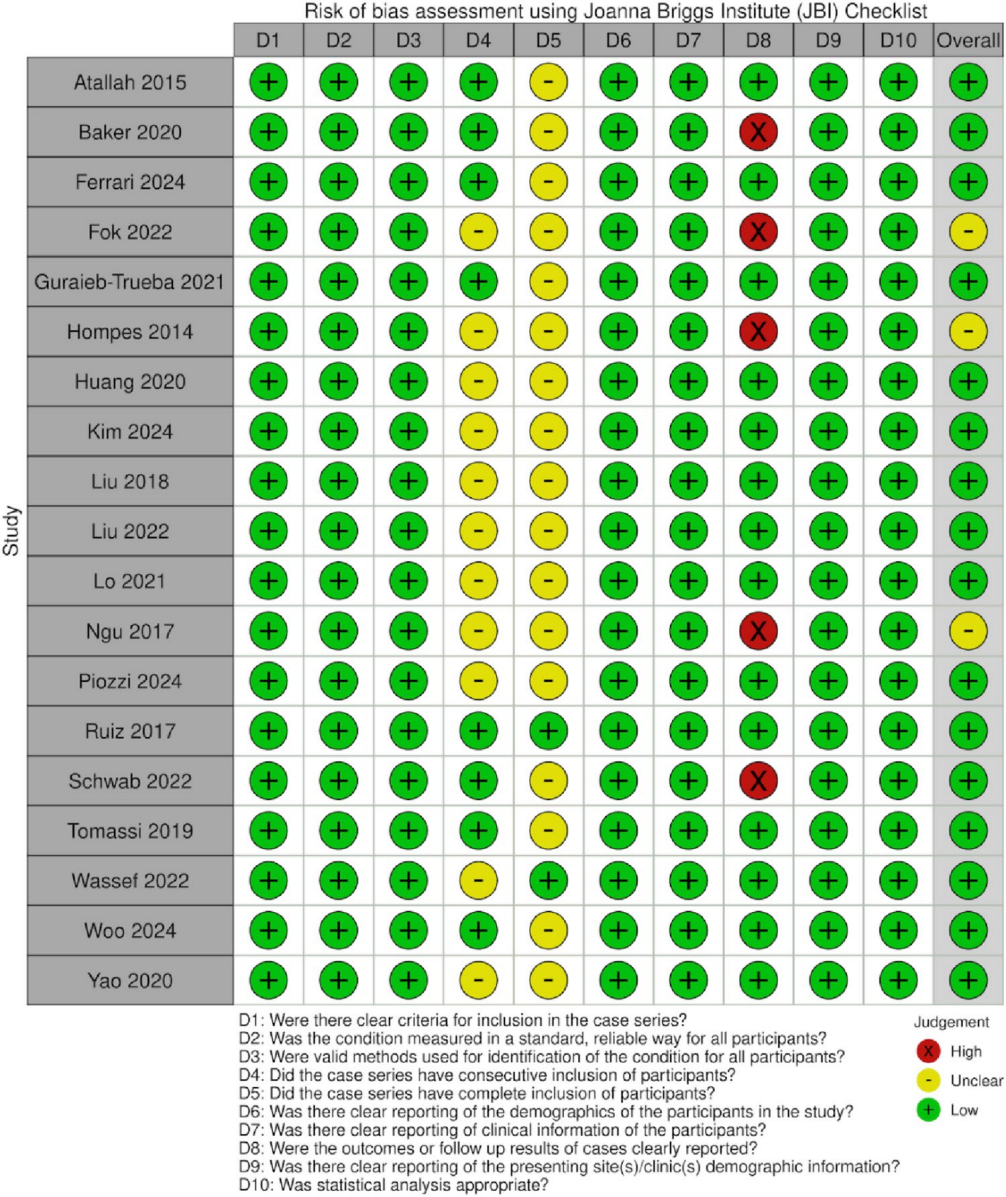
Joanna Briggs Institute (JBI) risk of bias assessment for case series

19 studies were assessed with Joanna Briggs Institute (JBI) Critical Appraisal Checklist for Case Series (Fig. 2), yielding overall scores between 7 and 9 out of 10. Criteria that were consistently well addressed across all studies included the use of clear inclusion criteria, standardized and reliable measurement of the condition among participants, comprehensive reporting of participant demographics and clinical information, and the application of appropriate statistical analyzes. Common limitations included incomplete detail on consecutive or complete inclusion of participants. An example of this is Atallah et al. [24] which stated that data was collected within a 33-month period but did not specify whether all eligible cases were included. Follow-up outcomes were not reported by five studies [12, 27, 29, 31, 38] Several other studies only reported a short follow-up period, but this was not scored negatively as the JBI domain only assessed whether follow-up was clearly documented, rather than duration. This included Huang et al. [32] and Piozzi et al. [36] who reported median follow up of 9.6 and 11.5 months respectively.

**Fig. 3:**
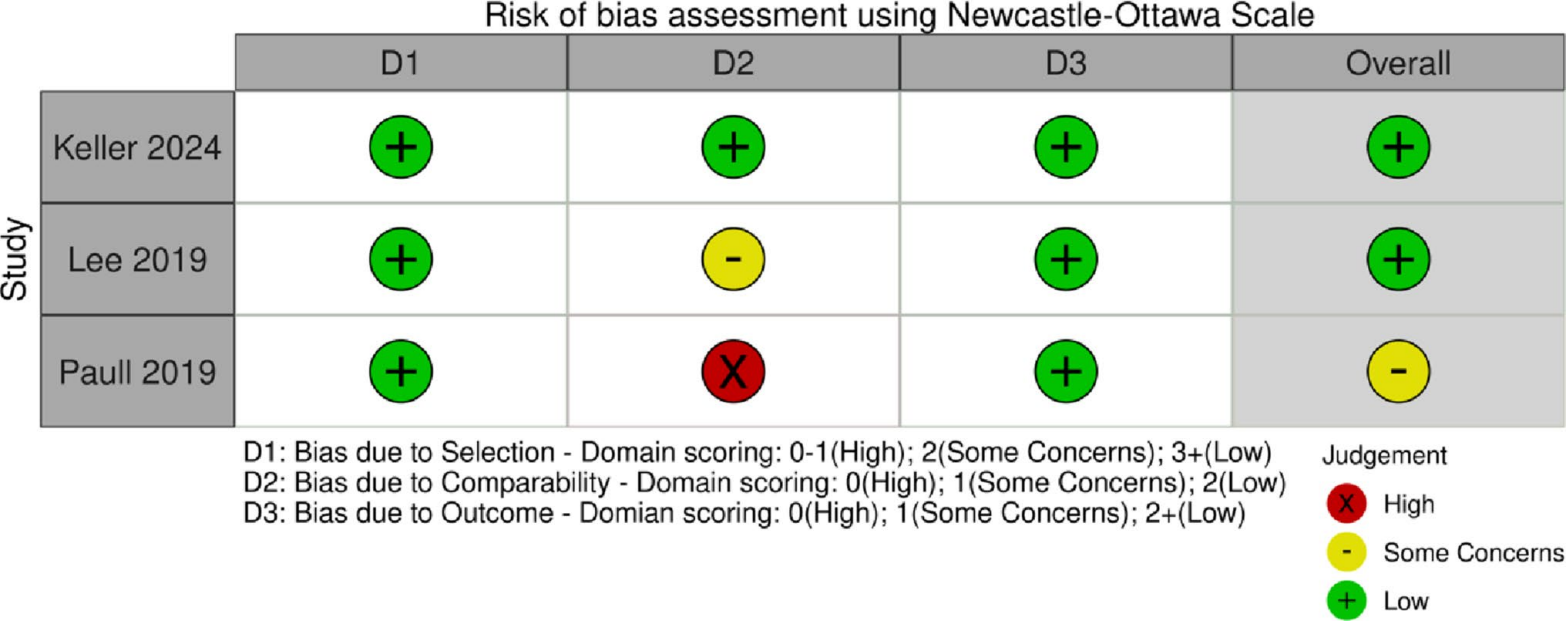
Newcastle-Ottawa Scale (NOS) risk of bias assessment for cohort studies

Three cohort studies were assessed using the Newcastle-Ottawa Scale (NOS) in Fig. 3. All studies received a score between 6 and 8 stars out of 9. Across the selection domain, every study had a clearly defined study population and exposure group. The study by Paull et al. [25] was rated as having a moderate risk of bias, due to its limitations in the comparability domain. Platform allocation was determined by availability rather than randomization, and all procedures were performed by a single surgeon, introducing potential operator bias. Outcome assessment was judged as low risk across all studies.

## Discussion

This systematic review analyzed 22 studies encompassing 437 patients. R-TAMIS demonstrated high rates of margin-negative resection, low perioperative morbidity and rapid postoperative recovery. Although heterogeneity in study design and reporting precluded formal meta-analysis, consolidating continuous variables through weighted pooling provided representative estimates of central trends. Collectively, the evidence suggests that R-TAMIS achieves oncological and perioperative outcomes comparable to laparoscopic TAMIS (L-TAMIS) and TEM.

Previous systematic reviews of R-TAMIS, provided important early insights [15, 16]. This review builds on those efforts by including an additional seven studies [20, 28, 30, 36, 38, 40, 41] with ≥ 5 patients, reporting detailed patient characteristics and operative, oncological and technical outcomes. By pooling descriptive weighted estimates, it provides more robust estimates of operative efficiency, recovery and oncological parameters, offering updated guidance for clinical practice and institutional planning.

The pooled descriptive weighted estimate operative time of 109 min in this review appears longer than the 76-minute mean reported for L-TAMIS [42]. Early comparative data from Lee et al. [10], found a median total operative duration of 102 min (IQR 22) for R-TAMIS vs. 100 min (IQR 55) for L-TAMIS (*p* = 0.70), indicating no significant difference. More recently, Schwab et al. [38] directly compared TEM, L-TAMIS and R-TAMIS in a single study, reporting median operative times of 110 min (IQR 78–136), 105 min (IQR 96–112) and 76 min (IQR 51–101) respectively with no statistically significant difference (*p* = 0.20). The earlier study [10] involved surgeons using both da Vinci Si and Xi platforms at a time when robotic integration was still emerging. As platforms evolved and surgical familiarity increased, later studies demonstrated meaningful efficiency gains. Tomassi et al. [13] reported a halving of mean operative time (38.7 min vs. 78.5 min, *p* = 0.00003) after transitioning from Si to Xi, and Paull et al. [25] observed shorter operative times (*p* = 0.055) and significantly reduced blood loss (*p* = 0.041) with the Flex Medrobotics platform. These findings suggest that refinements in robotic design and experience enable faster procedures.

Comparing TEM and R-TAMIS, Keller et al. [20] reported significantly shorter operative times for SPR-TAMIS (104 vs. 245 min, *p* = 0.027), likely reflecting improvements in robotic platform design and procedural technique. Another study [41] recorded one of the shortest docking durations, 9 min, using the da Vinci SP system. As noted by Friedman [43], SP deployment requires a minimum distance of ~ 7 cm, limiting its use for very proximal lesions. This aligns with the pooled mean tumor DAV in this review (8.3 ± 3.5 cm), comparable to L-TAMIS (7.18 cm, 0–20 cm) [5]. R-TAMIS also successfully managed lesions in the distal sigmoid (~ 25 cm), highlighting its extended reach with increased surgical competency [43].

R-TAMIS achieved high rates of margin-negative resection (96%, *n* = 393/408), mirroring L-TAMIS (96%) [42]. Postoperative morbidity was low: CD I–II events in 7%, D III in 1%, with only 2% requiring reoperation or readmission. These rates compare favorably with reported L-TAMIS morbidity of 18.5% [5], indicating equivalent safety despite R-TAMIS being a newer approach. Longer follow-up will also be needed to confirm recurrence rates, which were inconsistently reported but appear encouraging in the short term.

From a systems perspective, newer robotic platforms enable shorter docking and console times, combined with low morbidity and mean hospital stays under 2 days, potentially facilitating faster patient turnover and reduced bed occupancy. While R-TAMIS is associated with higher costs ranging from an additional € 1000 (~$ 1160) per procedure to total hospital costs approaching $ 10,000 [10, 31, 37, 40], Schwab et al. [38] reported no statistically significant difference in direct costs between TEM, L-TAMIS and R-TAMIS (median $ 6362, $ 6428 and $ 9226 respectively, *p* = 0.60), suggesting comparable expenditure. Efficiency gains in high-volume centers and multi-specialty use may further offset these costs. An additional direction to strengthen organ-preserving pathways is to couple transanal local excision with targeted mesorectal nodal assessment. Early technical reports describe combining local excision with fluorescence-guided mesorectal nodal sampling after ICG injection as a feasibility strategy to address the nodal staging gap without defaulting to TME [44].

The current evidence base is limited by small sample sizes, heterogeneity and a predominance of retrospective case series, with only one prospective study [27]. Given that R-TAMIS is an evolving technology with multiple platforms and iterative technical modifications, future evaluations should explicitly align with staged innovation frameworks (e.g., IDEAL) to strengthen comparability and facilitate progression beyond early case-series evidence [45]. Descriptive weighted estimates provided a useful overview but cannot eliminate inherent bias. Robotic platforms and patient positions varied across studies, and although this positional data was not extracted, this heterogeneity may influence operative outcomes, particularly operative durations. Only studies published in English were included, which may introduce language bias, and conference abstracts, preprints and other grey literature were excluded. Full-text peer-reviewed articles were the focus of the review to ensure complete reporting of relevant variables. Future work could focus on a single platform and standardized positioning to optimize R-TAMIS performance, as well as identify ideal patient selection. Beyond technical feasibility, a central determinant of suitability for organ-preserving transanal approaches remains confidence in nodal negativity; imaging-driven attempts to characterize mesorectal ‘sentinel’ distribution patterns have been proposed to refine this selection problem [46].

## Conclusion

R-TAMIS is a safe and feasible approach for local excision of early rectal tumors, achieving high rates of margin-negative resection with low perioperative morbidity and short hospital stay. Descriptive weighted estimates of available data suggest favorable operative efficiency and recovery profiles, but this interpretation is limited by study heterogeneity and the predominance of retrospective case series. Further prospective studies are needed to better define indications and comparative effectiveness across local excision platforms.

## Data Availability

No datasets were generated or analysed during the current study.
