# Correction: Robotic Transanal Minimally Invasive Surgery (R-TAMIS): A Systematic Review

**DOI:** 10.1007/s11701-026-03369-7

**Published:** 2026-04-21

**Authors:** Joachim Cheng En Ho, Clement Jing Cheng Yong, Aryan Raj Goel, Ayan Bin Rafaih, Irshad Shaikh, Muhammad Rafaih Iqbal

**Affiliations:** 1 College of Medicine and Veterinary Medicine, University of Edinburgh, Edinburgh, UK; 2 UCL Medical School, University College London, London, UK; 3 St Richard’s Hospital, University Hospitals Sussex NHS Foundation Trust, Chichester, UK; 4 Education Department, Aitchison College, Lahore, Pakistan; 5 Norfolk and Norwich University Hospitals NHS Foundation Trust, Norwich, UK; 6 University of East Anglia, Norwich, UK; 7 Mid and South Essex NHS Foundation Trust, Basildon, UK

**Correction to:** **Journal of Robotic Surgery** **(2026) 20:276**

10.1007/s11701-026-03229-4

In the original version of the article, the title was incorrectly given as “Robotic surgery (R-TAMIS): a systematic review” but should have been “Robotic Transanal Minimally Invasive Surgery (R-TAMIS): A Systematic Review”.

The original version of the article has been corrected.

